# miR-592 functions as a tumor suppressor in human non-small cell lung cancer by targeting SOX9

**DOI:** 10.3892/or.2021.8233

**Published:** 2021-11-25

**Authors:** Zhihong Li, Bai Li, Liang Niu, Liang Ge

Oncol Rep 37: 297-304, 2017; DOI: 10.3892/or.2016.5275

Following the publication of the above paper, the authors submitted a request to the Editorial Office to publish a corrigendum in light of a concern regarding potential contamination of their cells during the course of performing the experiments; at the same time, it was drawn to the Editors' attention by a concerned reader that certain of the colony-formation assay data shown in Fig. 2C were strikingly similar to data appearing in different form in another article by different authors. Owing to the fact that the contentious data in the above article were already under consideration for publication prior to its submission to *Oncology Reports*, the Editor has decided that this paper should be retracted from the Journal. After having been in contact with the authors, they agreed with the decision to retract the paper. The Editor apologizes to the readership for any inconvenience caused.

